# Carbon fiber–reinforced PEEK versus titanium implants: an in vitro comparison of susceptibility artifacts in CT and MR imaging

**DOI:** 10.1007/s10143-020-01384-2

**Published:** 2020-09-15

**Authors:** Theresa Krätzig, Klaus C. Mende, Malte Mohme, Helge Kniep, Marc Dreimann, Martin Stangenberg, Manfred Westphal, Tobias Gauer, Sven O. Eicker

**Affiliations:** 1 Department of Neurosurgery, University Medical Center Hamburg-Eppendorf, Martinistr. 52, 20246 Hamburg, Germany; 2 Department of Neuroradiology, University Medical Center Hamburg-Eppendorf, Hamburg, Germany; 3 Division of Spine Surgery, Department of Trauma and Orthopedic Surgery, University Medical Center Hamburg-Eppendorf, Hamburg, Germany; 4 Department of Radiation Oncology, University Medical Center Hamburg-Eppendorf, Hamburg, Germany

**Keywords:** Susceptibility artifacts, Imaging, Pedicle screws, Titanium, Carbon fiber–reinforced PEEK, Spine surgery

## Abstract

Artifacts in computed tomography (CT) and magnetic resonance imaging (MRI) due to titanium implants in spine surgery are known to cause difficulties in follow-up imaging, radiation planning, and precise dose delivery in patients with spinal tumors. Carbon fiber–reinforced polyetheretherketon (CFRP) implants aim to reduce these artifacts. Our aim was to analyze susceptibility artifacts of these implants using a standardized in vitro model. Titanium and CFRP screw-rod phantoms were embedded in 3% agarose gel. Phantoms were scanned with Siemens Somatom AS Open and 3.0-T Siemens Skyra scanners. Regions of interest (ROIs) were plotted and analyzed for CT and MRI at clinically relevant localizations. CT voxel–based imaging analysis showed a significant difference of artifact intensity and central overlay between titanium and CFRP phantoms. For the virtual regions of the spinal canal, titanium implants (ti) presented − 30.7 HU vs. 33.4 HU mean for CFRP (*p* < 0.001), at the posterior margin of the vertebral body 68.9 HU (ti) vs. 59.8 HU (CFRP) (*p* < 0.001) and at the anterior part of the vertebral body 201.2 HU (ti) vs. 70.4 HU (CFRP) (*p* < 0.001), respectively. MRI data was only visually interpreted due to the low sample size and lack of an objective measuring system as Hounsfield units in CT. CT imaging of the phantom with typical implant configuration for thoracic stabilization could demonstrate a significant artifact reduction in CFRP implants compared with titanium implants for evaluation of index structures. Radiolucency with less artifacts provides a better interpretation of follow-up imaging, radiation planning, and more precise dose delivery.

## Introduction

With the development of better diagnostic imaging and new targeted systemic therapies for different tumor entities, the incidence of spinal metastases in oncologic patients increases due to their prolonged survival [30]. Up to 40% of tumor patients develop metastases of the spine, with up to 10% becoming symptomatic and requiring surgery with decompression of the spinal cord and nerve roots and/or stabilization procedures often with a posterior screw-rod system. Those patients need close follow-up with regular computed tomography (CT) and magnetic resonance imaging (MRI) to assess local tumor control. Furthermore, the treatment paradigm for spinal metastases has changed over the last decades with the improvements made in radiation oncology from external body radiotherapy to stereotactic radiosurgery of the spine with good tumor control rates even in radio-resistant tumor entities [2, 19]. The surgical aim therefore is not necessarily to achieve maximal tumor resection for metastases anymore, but in many cases decompression of the spinal cord and nerve roots as well as stabilization procedures followed by external body radiotherapy or high-dose radiation treatment to achieve long-term progression free survival [18]. The standard implants for spine surgery, however, are made of titanium alloys, which produce relevant artifacts in CT and MRI making follow-up diagnostics and adjuvant radiotherapy with radiation planning and precise dose delivery difficult [1, 11, 16].

Polyetheretherketon (PEEK) is a radiolucent biomaterial which started to replace metal implants in orthopedics and trauma since the 1990s and is also used for interbody fusion cages in spine surgery for several years [17, 26, 33]. Over 15 years ago, pedicle screws were started being produced from carbon fiber–reinforced polyetheretherketon (CFRP) coated with porous titanium for better osseointegration. Those special screws are in clinical use with good mechanical stability and present an excellent alternative to titanium in order to reduce imaging artifacts [10, 21, 28]. Approval of the American Food and Drug Administration (FDA) was obtained recently in 2019. Until now, several studies evaluated the advantages in imaging of these new implants or concentrated mainly on the advantages in radiation oncology according to improved radiation planning as well as dose calculation and distribution [4, 10, 23, 28]. In all studies, the CFRP screws were implanted in vivo in human subjects. As the protocols hold various interindividual differences such as different implantation angles or different distances of the screws, different implant diameters and sizes as well as soft tissue variabilities in the subjects, and restlessness of the patient during the examination, a distinct comparison between titanium and CFRP implants and a reliable quantitative analysis is not possible. We therefore analyzed susceptibility artifacts in CT and MR imaging of standardized titanium and CFRP screw-rod constructs for posterior spinal stabilization in agarose gel phantoms in vitro.

## Material and methods

### Phantoms

**Fig. 1:**
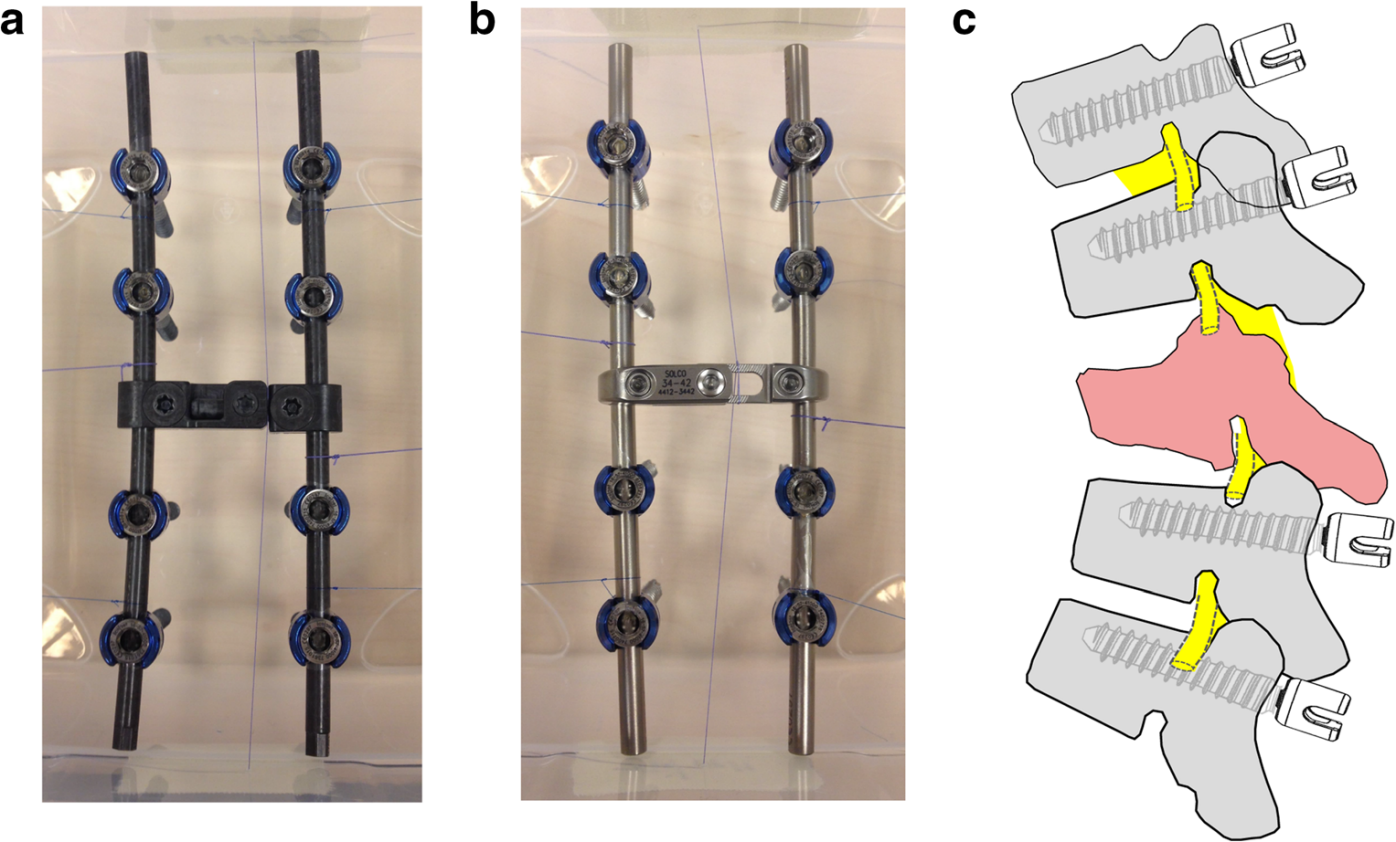
Construct design. **a** Picture representing the carbon fiber–reinforced PEEK (CFRP) construct with eight pedicle screws (5.5 × 40 mm), two 160-mm rods, and a cross-link. **b** Analogous picture for the full titanium construct using 5.5 × 40 mm pedicle screws, two titanium 160-mm rods, and a cross-link. **c** Schematic representation of a sagittal profile of the thoracic spine with a tumor-infiltrated vertebra in the middle (red vertebra). Instrumentation covers two vertebrae above and below

Single full titanium or CFRP pedicle screws and fixed constructs with eight pedicle screws (four screw pairs), two rods and one cross-link, either titanium or CFRP, were separately embedded in either 1800 cm^3^ (screws only) or 4600 cm^3^ (constructs) of 3% agarose gel to guarantee maximum comparability. We used either CFRP pedicle screws (BlackArmor®, icotec AG, Altstaetten, Switzerland) coated with radiolucent porous titanium for enhanced osseointegration, in which only the tulip and set screw are made from titanium. A tantalum marker is placed in the tip of the CFRP screw shank for intraoperative visualization. The other construct was made of full titanium pedicle screws. All screws used had a diameter of 5.5 mm and 40-mm length. Rods (length: 160 mm) and cross-links were either completely made of CFRP or titanium. CFRP consists of 55 vol% carbon embedded in a polyetheretherketon matrix. Screws were placed as shown in Fig. 1 with the same distance on each side of 3 cm in both constructs, mimicking a thoracic instrumentation including two vertebrae above and below the tumor skipping the tumor infiltrated vertebra, where the distance of the screws was 5 cm.

### Imaging

Gel phantoms were scanned by a CT scanner (Somatom Definition AS Open, Siemens Healthcare GmbH, Erlangen, Germany) and a 3.0-Tesla (T) MR scanner (Skyra, Siemens Healthcare GmbH, Erlangen, Germany). Standard clinical protocols for spine imaging of our institute were used for each modality, and the phantoms were scanned in transversal and sagittal orientation. All phantoms were placed in the same position and level for all scans to ensure reproducibility and maximum comparability.

CT scans were performed using spiral technique with 120 kV and 110 mAs, 512 × 512 matrix, pixel spacing = 0.9 mm, slice thickness = 1 mm, and convolution kernel H31s.

The MR imaging protocol consisted of the following sequences: (a) 2D T1-weighted turbo spin echo (TSE) sequence in sagittal plane with TE = 8.5 ms, TR = 400 ms, 17 slices with slice thickness 3 mm, pixel spacing = 0.729 mm, FOV = 280 mm × 280 mm, and bandwidth = 260 Hz/pixel; (b) 2D T1-weighted turbo spin echo (TSE) sequence in sagittal plane with slice encoding for metal artifact correction (SEMAC), TE = 7.9 ms, TR = 800 ms, 17 slices with slice thickness = 3 mm, pixel spacing = 0.875 mm, FOV = 280 mm × 280 mm, and bandwidth = 680 Hz/pixel; (c) 2D T2-weighted turbo inversion recovery magnitude (TIRM) sequence in sagittal plane with echo time TE = 44 ms, inversion time TI = 220 ms, repetition time TR = 4000 ms, flip angle = 160°, 17 slices with slice thickness = 3 mm, pixel spacing = 1 mm, field of view FOV = 320 mm × 320 mm, and bandwidth = 250 Hz/pixel; and (d) 2D T2-weighted short TI inversion recovery (STIR) sequence in sagittal plane with slice encoding for metal artifact correction (SEMAC), TE = 38 ms, TI = 220 ms, TR = 4000 ms, flip angle = 140°, 17 slices with slice thickness = 3 mm, pixel spacing = 0.625 mm, FOV = 320 mm × 320 mm, and bandwidth = 675 Hz/pixel.

For CT analyses, a linear region of interest (ROI) for each image was defined across the center of the biomaterial in the respective orientation, size, and slice in the transverse direction parallel to the longitudinal axis of the implants within clinical relevant regions, i.e., the spinal canal and the posterior and the anterior parts of the vertebral body (Fig. 3a). Only the neuroforamen was evaluated in the sagittal slices (Fig. 3b). A signal intensity profile was calculated to evaluate the susceptibility artifact produced by each material. The intensity profile of the baseline was determined on a control phantom with only 3% agarose gel by using the same procedure. Susceptibility artifacts were extracted from the defined ROIs, measured as voxel gray scales in Hounsfield units (HU).

For MRI signal voids around the screws on T1 TSE, T1 SEMAC, T2 TIRM, and STIR SEMAC, images were measured by different observers (KCM, TK, MM, MS) according to their length and diameter. Those measurements including the artifacts around the implants were then correlated with the original screw size.

### Statistical analysis

Statistical analysis and graph layout were performed using GraphPad Prism 8. Graphical analysis was performed using FIJI [29], and rectangular regions of interest for CT data comparison were defined for the spinal canal (22 × 15 mm, box 1), the posterior margin of the vertebra (20 × 15 m, box 2), the anterior margin of the vertebra (18 × 10 mm, box 3), and the neuroforamen (sag. CT, 20 mm × 10 mm, box 4) (Fig. 3 a and b). Boxes 1–3 were measured between each four screw pairs of one construct. Box 4 represents the measurement of the neuroforamen and therefore consists of three measurements between the screws in sagittal plane of the CT scan. ROIs therefore represent four (boxes 1–3) or three (box 4) measurements, respectively (Mann-Whitney *U* test). Profile plots of Hounsfield units (4-cm width and 1-cm height) and MRI density values were recorded for each screw (CT: conventional and iterative metal artifact reduction (IMAR) program; MRI: T1 TSE, T2 TSE, STIR SEMAC, T1 SEMAC) and displayed for graphical comparison. The “real” screw size is displayed in comparison with the signal alteration in MRI and CT. For statistical analysis, we decided to use only the CT density profiles due to the standardization of Hounsfield units in CT measurements using one-way ANOVA with post hoc Tukey test for multiple comparisons.

## Results

**Fig. 2:**
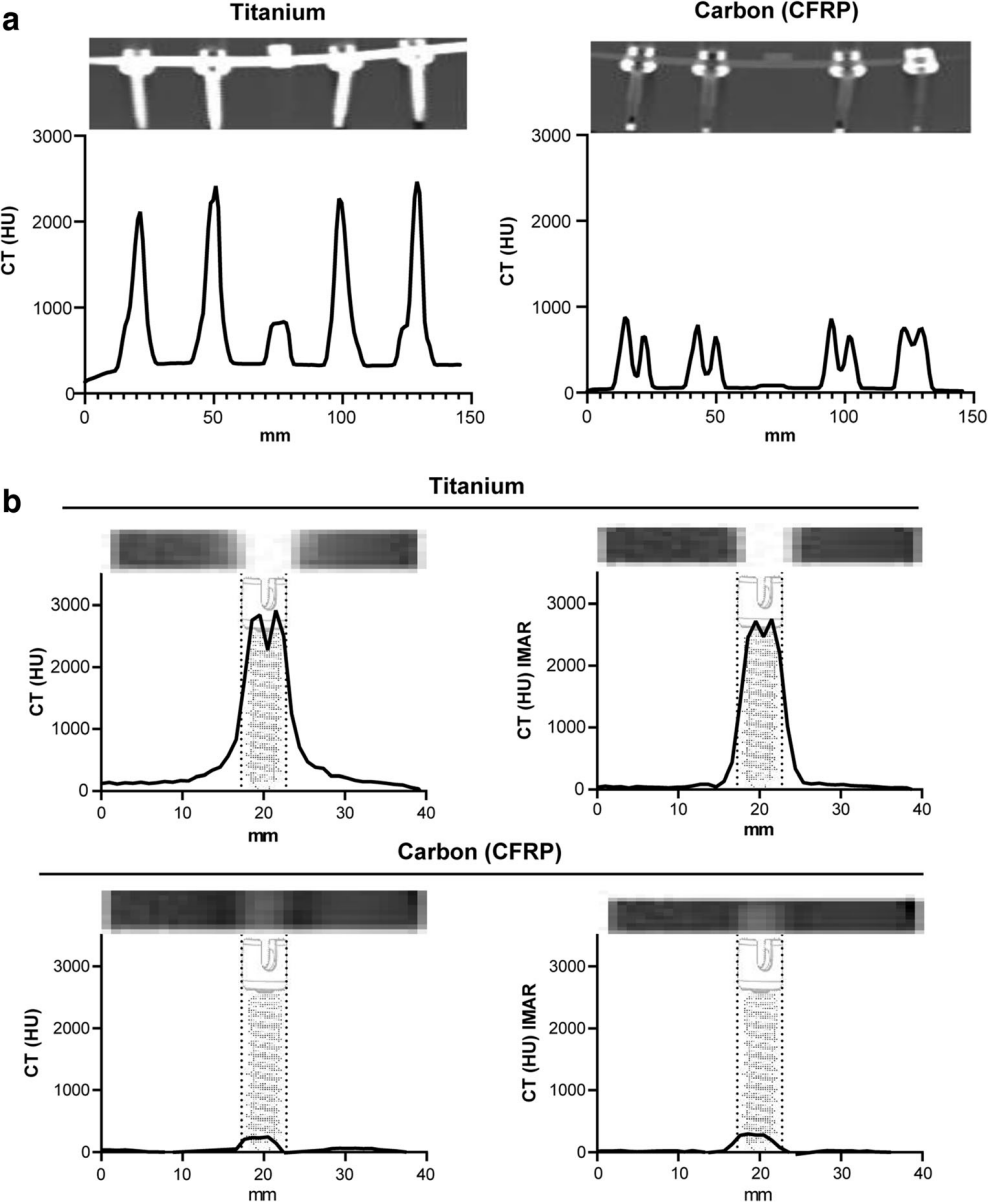
CT measurements. **a** Sagittal overview and measurement of the whole screw-rod construct. Left panel shows the full titanium construct. Right panel shows the carbon fiber–reinforced PEEK (CFRP) construct. Above each graph, conventional CT scan in sagittal plane of the relative construct phantom is shown. HU = Hounsfield Units. **b** Measurements of CT density (HU) for single screws in conventional CT imaging (left panels) and iterative metal artifact reduction (IMAR) images (right panel) with a section of the CT scans of the screws showing the relative susceptibility artifacts above the graphs. Measurements including the whole construct (**a**) were performed on four screws and the rod. Measurements for single screws (**b**) included one screw with three measurements

Single screws and screw-rod phantoms (Fig. 1) were evaluated in CT and MRI. The constructs showed high HU intensities for the titanium construct in sagittal 685.1 ± 587.3 HU (ti) vs. 223.4 ± 251.0 HU (CFRP, *p* < 0.001) as well as in axial CT images. In the CFRP constructs, most of the intensity changes were seen at the screw tulip (1192.0 ± 664.3 HU (ti) vs. 564.3 ± 171.6 HU (CFRP), *p* < 0.001) which is still made of titanium and the cross-link (714.7 ± 157.4 HU (ti) vs. 81.3 ± 5.4 HU (CFRP) *p* < 0.001) (Fig. 2a). Single screw ANOVA of density profile plots of the screw diameter (5.5 × 40 mm) in the conventional CT showed significant differences between single CFRP and titanium screws with 47.64 ± 94.86 HU (mean ± SD) for CFRP vs. 317.7 ± 42.43 HU for titanium (*p* < 0.001). For IMAR CT, there was also a significant difference between CFRP with 46.17 ± 89.66 HU compared with titanium with 347.8 ± 136.9 HU (*p* < 0.001) (Fig. 2b).

**Fig. 3:**
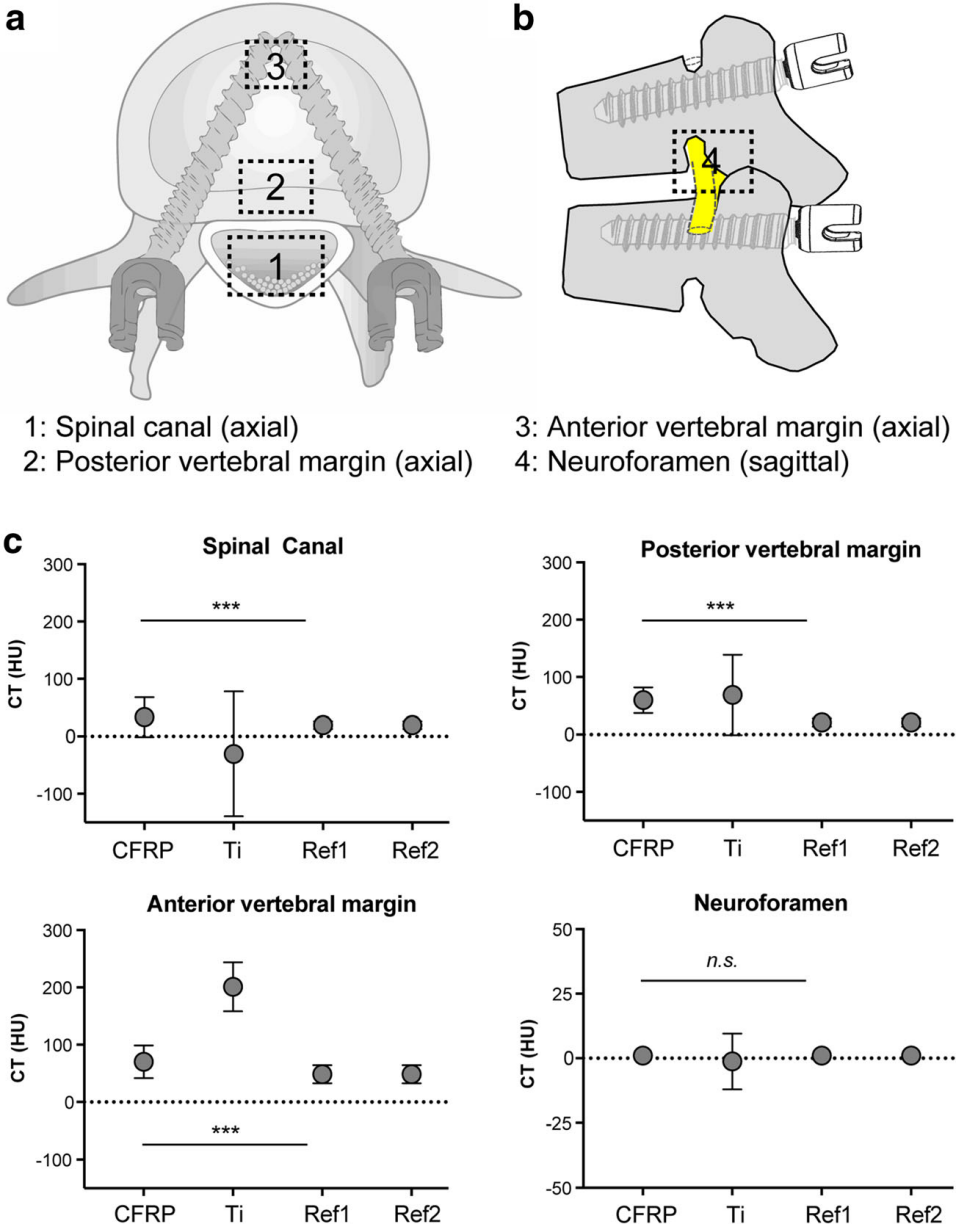
Region of interest (ROI) definition for CT scans and comparison of HU. **a** Localization of ROIs for CT measurements. Measurements of the boxes 1–3 were performed on axial images with a box size of 22 × 15 mm for the spinal canal (1), 20 × 15 mm for the posterior margin of the vertebra (2), and 18 × 10 mm for the anterior margin of the vertebra (3). **b** Measurements for the neuroforamen (4) were performed on sagittal images with a box size of 20 × 10 mm. **c** Comparison of CT densities in HU for the defined ROIs with *** = *p* < 0.001 and n.s., not significant. Ref1, reference 1 and Ref2, reference 2 are the densities in HU of the agarose gels in the boxes of the CFRP constructs and the titanium constructs, respectively

Construct evaluation of the key ROIs (Fig. 3a and b) showed significant differences for density values at the area of the spinal canal with − 30.7 ± 108.8 HU (mean ± SD) for titanium vs. 33.4 ± 34.6 HU for CFRP (*p* < 0.001), at the posterior margin of the vertebra with 68.88 ± 69.92 HU (ti) compared with 59.75 ± 22.34 HU (CFRP) (*p* < 0.001), and at the anterior part of the vertebra with 201.2 ± 42.7 HU (ti) vs. 70.4 ± 28.4 HU (CFRP) (*p* < 0.001), respectively. No significant difference could be detected at the area of the neuroforamen with − 1.3 ± 10.8 HU for titanium compared with 1.0 ± 0.0 HU for CFRP (ns) (Fig. 3c). Densities in HU of the agarose gel in the CFRP box (reference 1) and the titanium box (reference 2) worked as references.

**Fig. 4:**
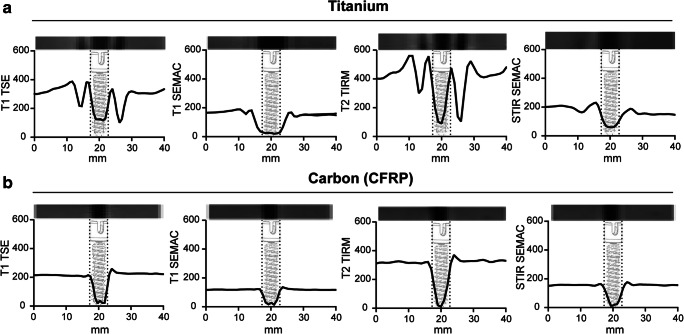
MRI measurements. **a** Titanium and **b** carbon fiber–reinforced PEEK screw measurements in different sequences of magnetic resonance imaging (MRI): T1 turbo spin echo (TSE), T1 slice encoding for metal artifact correction (SEMAC), T2 turbo inversion recovery magnitude (TIRM), and short tau inversion recovery (STIR) SEMAC. Above all graphs, a section of the MRI scan of the screws is shown with the relative susceptibility artifacts

MRI data is presented in Fig. 4. All observers stated better visualization with less intensity changes of the surrounding tissue and ROIs for CFRP screws in all sequences T1 TSE, T2 TSE, STIR SEMAC, and T1 SEMAC. Screw length and diameter were measured a lot closer to the original size in MRI with CFRP constructs compared with titanium phantoms. Analysis of the MRI scans (Fig. 4) was performed with only one measurement for each sequence as not all sequences were available in compared section thicknesses and orientations. Due to *n* = 1, no statistical analysis was performed.

## Discussion

Previous studies analyzed artifacts of titanium versus CFRP implants in spine surgery either in vivo or related to radiation planning only [4, 28]. We used a typical implant configuration for thoracic stabilization in patients with one level vertebral metastasis in a gel phantom to avoid interindividual differences in humans, such as different soft tissue depths and different implant locations, screw angles, and artifacts due to motion during the scan. In our study, we could demonstrate a significant artifact reduction in CFRP implants compared with titanium implants for evaluation of index structures as the spinal cord and vertebral body.

It was shown many years ago that PEEK is a biologically compatible and inert semi-crystalline thermoplastic polymer with minimal toxicity, exhibiting good mechanical and chemical properties, such as high strength, good wear resistance, and fatigue properties. It was initially introduced in spinal surgery in the form of interbody cages also in the form of carbon fiber–reinforced PEEK [3, 5, 17]. Since 2007, PEEK rods are introduced for dynamic stabilization [12, 17, 24]. In terms of stiffness and resistance to motion, several cadaver studies could show that PEEK or CFRP rods were comparable with the stability of titanium rods [7, 27]. Lindtner et al. could also demonstrate in their biomechanical study that CFRP pedicle screws implanted in cadaveric lumbar vertebrae have a similar screw anchorage than titanium screws on the contralateral pedicle [21]. Osseointegration is improved by thin titanium coating of the implants, although it has to be stated that the fracture strain can be influenced by the coating process [9, 32]. Overall CFRP is considered comparable with titanium according to its use in stabilization procedures in orthopedic, trauma, and spine surgery [6, 17]. A striking advantage of this material is its radiolucency at the standard radiograms which makes it also barely visible on CT and MRI allowing easier detection of local recurrence as well as better radiation planning. Furthermore scattering effects during radiotherapy can be avoided [4, 13].

This study to our knowledge first describes qualitatively and quantitatively the differences of identical titanium and CFRP screw-rod systems for the spine without the interindividual differences going along with analyses in vivo. In CT imaging, we could show significant differences (*p* < 0.001) in our titanium and CFRP phantoms for different ROIs in both conventional scans and even the IMAR program, which already creates considerably less artifacts. As demonstrated in our first experience with CFRP screws in vivo, we can now confirm an excellent visualization of the area adjacent to the implants [10]. The baseline of the agarose gel was the same in both boxes. At the anterior part of the virtual vertebra, CFRP and titanium screws both showed higher HU than at other parts of the screw shank. Still, evaluation of the surrounding bone and tissue was significantly better in CFRP than titanium constructs for that ROI. This indicates that the configuration of the screw tip itself holds for more artifact generation in comparison with the remaining screw shank and is not only due to the tantalum marker placed in the tip of the CFRP screw for better visualization during implantation. The not significant difference between titanium and CFRP implants for the area of the neuroforamen, measured on sagittal slices, might be explained by a different angle in the application of the x-ray beams causing less overall scattering. Additionally, screw tulips made of titanium in both screw materials are located more lateral than the spinal canal near the neuroforamen and might add to more artifact generation as well. Spine tumors, however, making stabilization necessary are mainly located in the vertebra itself only affecting the dorsal structures as neuroforamen and spinal canal secondarily [19, 30]. Nonetheless, screw tulips made of CFRP would be desirable for further improvement of follow-up imaging and radiation planning where especially the neural structures have to be spared. A better visualization of the neural structures with emphasis on the neuroforamen could also extend the indication for CFRP screws on degenerative cases. In MR imaging were, even in the artifact reduced programs, as demonstrated earlier in other in vivo studies, less artifacts detectable around the CFRP material in all diagnostically relevant regions compared to the titanium phantoms [10, 28].

Our study is of clinical relevance as especially over the last decades, the paradigm in treating spine metastases has changed and radiation therapy is one of the main treatment options in cancer therapy. The surgical procedures are more and more tailored to the patients, their overall constitution and the aim of preserving a long-lasting good neurological and physical condition. This often leads to a smaller surgery with following radiation therapy, also in presumed radio-resistant tumor entities [2, 19, 30]. However, accuracy and resolution in pre-treatment three-dimensional CT imaging is crucial for the correct planning, especially according to the delineation of the target volume and organs at risk near the treatment area as well as for an exact dose calculation and optimization of beam intensities [11]. In the presence of metal implants in the field of view of a CT scanner, image artifacts are created due to beam hardening, scatter, and noise [1, 22]. In previous studies, it could be shown that irradiation foci and dose distribution were easier and more precise to specify in CFRP implants compared with the radiation planning with present metal artifacts, which we can support with our quantitative analysis [10]. Especially in proton and ion radiotherapy, metallic implant materials interfere with exact radiation planning and dose distribution and should be avoided [14, 15]. Two study groups even reported a 5 to 10% radiation dose reduction to tissues in regions behind titanium rods due to the attenuation effect. Chordoma patients who received proton therapy showed significant association of reduced tumor control or increased local recurrence when titanium-based surgical stabilization was involved [8, 20, 25, 31].

The titanium coating of the CFRP screws, however, had a negligible effect on the dose distribution in a study by Nevelsky and colleagues who compared overdose and underdose due to backscatter and attenuation in titanium screws with CFRP screws with and without titanium coating [23]. We did not use CFRP screws without titanium coating in our agarose phantom as Devine and colleagues could show a significantly higher bone apposition in CFRP implants with titanium coating and we only used material which is already approved for clinical use [9]. In our study, we could confirm the significantly less artifact production in the CFRP implants compared with conventional titanium constructs.

The main limitation of our study is the lack of statistical analysis for MRI due to the low sample size. Images could have been evaluated additionally by different radiologists according to the impairment of visualization of ROIs due to artifacts, but these judgments would have been highly subjective as well. Another limitation of our study, which was however part of the protocol, is the lack of different tissue densities. A following project with phantoms embedded in bone, muscle, fat, cerebrospinal fluid, etc. could help to assess differences in imaging qualities even better.

## Conclusion

We could demonstrate a significant artifact reduction in CFRP implants compared with titanium implants in CT scans for evaluation of index structures as spinal cord and the vertebra in an agarose gel phantom model. Radiolucency with less artifacts provides a better interpretation of follow-up imaging, radiation planning, and a more precise dose delivery to the target area in radiation therapy, rendering CRFP implants superior to titanium implants in patients with spinal tumors needing stabilization.

## Data Availability

All relevant data are included in the manuscript. Additional data and information will be provided from the corresponding author upon reasonable request.
